# A Progress Report of the IFCC Committee for Standardization of Thyroid Function Tests

**DOI:** 10.1159/000358270

**Published:** 2014-05-07

**Authors:** Linda M. Thienpont, Katleen Van Uytfanghe, Sofie Van Houcke, Barnali Das, James D. Faix, Finlay MacKenzie, Frank A. Quinn, Michael Rottmann, Annick Van den Bruel, for the IFCC Committee for Standardization of Thyroid Function Tests (C-STFT)

**Affiliations:** ^a^Laboratory for Analytical Chemistry, Faculty of Pharmaceutical Sciences, Gent University, Gent, Bruges, Belgium; ^b^Department of Endocrinology, General Hospital Sint Jan, Bruges, Belgium; ^c^Biochemistry and Immunology Laboratory, Kokilaben Dhirubhai Ambani Hospital and Medical Research Institute, Mumbai, India; ^d^Stanford University School of Medicine, Palo Alto, Calif., USA; ^e^Medical and Scientific Affairs, Abbott Diagnostics, Abbott Laboratories, Abbott Park, Ill., USA; ^f^Birmingham Quality/UK NEQAS, University Hospitals Birmingham NHS Foundation Trust, Birmingham, UK; ^g^Roche Diagnostics GmbH, Penzberg, Germany

**Keywords:** Free thyroxine, Thyrotropin, Standardization, Harmonization, Method comparison, Traceability

## Abstract

**Background:**

The IFCC Committee for Standardization of Thyroid Function Tests aims at equivalence of laboratory test results for free thyroxine (FT4) and thyrotropin (TSH).

**Objectives:**

This report describes the phase III method comparison study with clinical samples representing a broad spectrum of thyroid disease. The objective was to expand the feasibility work and explore the impact of standardization/harmonization in the clinically relevant concentration range.

**Methods:**

Two sets of serum samples (74 for FT4, 94 for TSH) were obtained in a clinical setting. Eight manufacturers participated in the study (with 13 FT4 and 14 TSH assays). Targets for FT4 were set by the international conventional reference measurement procedure of the IFCC; those for TSH were based on the all-procedure trimmed mean. The manufacturers recalibrated their assays against these targets.

**Results:**

All FT4 assays were negatively biased in the mid- to high concentration range, with a maximum interassay discrepancy of approximately 30%. However, in the low range, the maximum deviation was approximately 90%. For TSH, interassay comparability was reasonable in the mid-concentration range, but worse in the pathophysiological ranges. Recalibration was able to eliminate the interassay differences, so that the remaining dispersion of the data was nearly entirely due to within-assay random error components. The impact of recalibration on the numerical results was particularly high for FT4.

**Conclusions:**

Standardization and harmonization of FT4 and TSH measurements is feasible from a technical point of view. Because of the impact on the numerical values, the implementation needs careful preparation with the stakeholders.

## Introduction

It is generally accepted that laboratory medicine should focus on improving consistency and reproducibility of measurements across different laboratories and methods [1,2]. Immediate benefits to expect from accomplishing this goal are easy transfer and/or combined analysis of laboratory data, significant reduction of repeated measurements, and increased confidence in patient care. In the mid- to long term, development of evidence-based practice guidelines with recommendations for uniform instead of assay-specific decision limits would become possible. The process to achieve this goal is called ‘standardization’ and makes use of a reference measurement system [3]. Typical examples of internationally standardized laboratory tests include lipids, HbA1c, creatinine, and enzymes [4,5,6,7], but more are under development [8,9,10,11].

The IFCC Committee for Standardization of Thyroid Function Tests (C-STFT) aims at equivalence of laboratory testing for total and free thyroid hormones and thyrotropin (TSH) [12,13,14]. The committee was established in light of the prevalence of thyroid disease, the frequency of laboratory testing, and multiple reports on discrepant measurement results. For total thyroxine/triiodothyronine testing, a state-of-the-art SI-traceable reference measurement system was already available for implementation [14]. For free thyroid hormones and TSH, the C-STFT had to start the process from scratch. The measurands needed to be defined and reference measurement procedures or a valid alternative developed. For free thyroxine (FT4), this led to an IFCC-approved international conventional reference measurement procedure (cRMP), based on equilibrium dialysis isotope dilution-liquid chromatography-tandem mass spectrometry (ED ID-LC/tandem MS) [15]. In the case of TSH, for which it is technically unlikely to have an RMP available in the mid- to short term, the C-STFT proposed a statistical alternative for ‘harmonization’ rather than standardization [16,17].

With these two tools, the committee developed a strategy to assess the quality and comparability of FT4 and TSH assays, followed by investigation of the feasibility of standardization/harmonization. This was done in cooperation with the in vitro diagnostic (IVD) industry and in essence used several method comparison studies with panels of single-donation sera [12,13,18]. These showed the assays were of good quality, but confirmed considerable interassay difference in results. Simultaneously, they demonstrated the feasibility of aligning the assays. However, the conducted studies had one important limitation: they only used samples from apparently healthy donors and few samples from patients with thyroid disease. Therefore, C-STFT performed a phase III method comparison study using a wide variety of samples from patients with thyroid disease. This allowed the exploration of the impact of standardization/harmonization across the clinically relevant concentration range. This was particularly crucial for TSH in view of the evidence for thyroid disorder-specific glycoforms, implying that antibodies in current assays may either demonstrate glycosylation-specific binding or are ‘glycosylation blind’ [19,20].

## Materials and Methods

### Panels of Clinical Samples

Serum samples (74 for FT4, 94 for TSH) were obtained from commercial sources (SLR Research Corporation, Carlsbad, Calif., and PromeddX, Norton, Mass., USA) working with clinical centers, and by courtesy of endocrinologists. All collection centers had the approval of their ethical review boards. The two serum panels were selected to represent the relevant clinical measurement range (see table 1 for the inclusion and exclusion criteria).

### Study Participants

Eight IVD manufacturers participated, with a total of 13 assays for FT4 and 14 for TSH (table 2). It is important to note that the assays are randomly coded, without a logical relationship between the FT4 and TSH assays (for the rationale, see online suppl. material; www.karger.com/doi/10.1159/000358270).

### Target Setting of the Samples

FT4 target values were assigned at Ghent University with the ED ID-LC/tandem MS cRMP [15]. The method's expanded uncertainty of measurement (k = 2, 95% confidence level) according to the measurement protocol used (3 replicates, in independent runs) is estimated at 7.6%. The targets for TSH were based on the all-procedure trimmed mean (APTM) inferred by an iterative calculation process with adaptation of assay-specific outliers (see online suppl. material).

### Method Comparison Study and Recalibration

The routine assays performed duplicate measurements within one run with one reagent lot, and under internal quality control conditions selected by the respective IVD manufacturers. To assure random distribution of sample concentrations, the replicates were measured in upward and downward sequences. The manufacturers included their master calibrators for use in subsequent recalibration.

### Data Analysis

#### Concentration range

For FT4, one sample with a concentration below the limit of quantitation of the cRMP (at 0.86 pmol/l) was excluded from data analysis; the same applied for 7 TSH samples with concentrations below the typical functional sensitivity limit (0.012 mIU/l) because 3-6 assays did not report results.

#### Assay-Specific Outliers

Assay-specific outliers were identified from inspection of the scatter and difference plots (absolute, %-difference, and %-residuals) of the means of duplicates against the FT4 cRMP target values and the first APTM in the iterative process, and from the %-difference between the duplicates and the interassay CV observed for the samples. Outlying results were substituted with values that fit best in the %-residual plot, whereby both replicates were given the same value. This prevented that (1) the APTM for that sample would be biased and (2) information for the recalibration exercise would be lost. Because the outlier-substituting process changed the initially calculated APTM, it was iterated until the final one was reached. The limit used for outlier detection corresponded to 3 standard deviations. For FT4, 11 outliers from a total of 923 data were identified and adapted; for TSH, there were 29 out of 1,218. Outliers were excluded from calculation of the within-run coefficient of variation and between-run differences.

#### Status of Standardization/Harmonization before and after Recalibration

The status of standardization and the effect of recalibration by the IVD manufacturers based on their master calibrators were investigated from the assay biases (%; mean of duplicates) for each of the samples compared to the respective targets. Also the mean bias (%) of each assay relative to the mean target was assessed, i.e. by averaging the individual %-difference of the duplicate means from their respective target values. This was done for a low (FT4: <9 pmol/l; TSH: 0.03-0.5 mIU/l), mid- (FT4: 9-27 pmol/l; TSH: 0.5-5 mIU/l), and high (FT4: >27 pmol/l; TSH: >5 mIU/l) concentration range. Furthermore, the interassay variation for the individual samples (‘interassay CV’) before and after recalibration was evaluated. It was calculated from the ratio between the standard deviation on each assay's results per sample and the mean sample concentration (FT4) or APTM (TSH). For statistical analysis and plotting, Microsoft Excel® 2010 was used.

## Results

### Concentration Ranges Covered by the Clinical Samples

The clinical samples adequately addressed concentrations typical for hypo-, eu-, and hypothyroidism, i.e. ranging from 3 to 77 pmol/l for FT4 (cRMP targets), and from 0.04 to 80 mIU/l for TSH (APTM) [21,22].

### Status of Standardization before and after

**Fig. 1:**
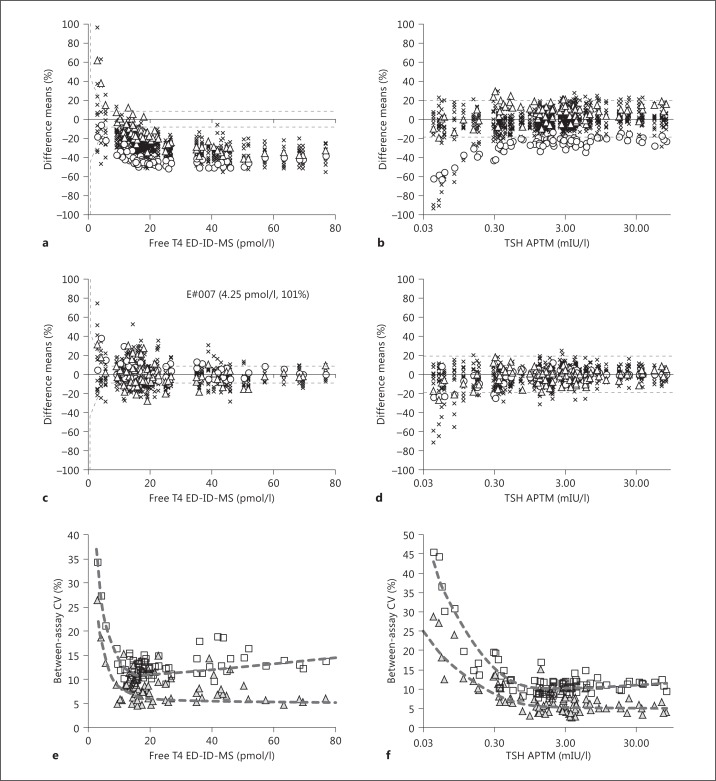
**a-d** Plots showing the %-difference before (FT4: **a**, TSH: **b**) and after recalibration (FT4: **c**, TSH: **d**). The most discrepant assays before recalibration are highlighted by special symbols (FT4: K, circles; M, triangles; TSH: I, circles; K, triangles); all other assays are indicated with the same symbol X. **e**, **f** Interassay CV (FT4: **e**, TSH: **f**) before (squares) and after (triangles) recalibration by IVD manufacturers.

Figure 1A and table 3 show that, compared to the cRMP targets, all FT4 assays were strongly negatively biased (beyond the arbitrary limit of −10%) for concentrations >27 pmol/l (median bias: −37%, range: −21 to −48), and between 9 and 27 pmol/l (median bias: −24%, range: −14 to −42). In contrast, they were negatively as well as positively biased in the range <9 pmol/l (median bias: −9%, range: −28 to 62). IVD manufacturers were able to eliminate the observed bias of their assays by recalibration to the cRMP target values (fig. 1c).

Figure 1b and table 3 demonstrate that for TSH, the assays' mean bias to the APTM was slightly more negative in the low concentration range (0.03-0.05 mIU/l; median bias: −6%; range: −33 to 12) than in the mid-range (0.05-5 mIU/l; median bias: −2%; range: −23 to 11) and >5 mIU/l (median bias: −0.3%; range: −21 to 12). The number of TSH assays outside the ±10% limit around the APTM was between 2 and 5 out of 14, dependent on the concentration range. After recalibration to the APTM, the distribution of the differences around zero became more symmetric (fig. 1d).

The effect of recalibration to the respective targets can also be inferred from the decrease in interassay CVs, which ranged from 9.7% (mid-concentration range, before recalibration) to 3.4% (after) for FT4 (fig. 1e); for TSH it ranged from 9.1% (also mid-range) to 5.9% (fig. 1f).

## Discussion

C-STFT conducted this phase III method comparison study for FT4 and TSH to reconfirm the feasibility and investigate the impact of standardization/harmonization on a clinically relevant concentration range. The emphasis was on remediating limitations of previous studies by using samples representing a broad thyroid spectrum and sourced from clinical settings. However, samples from patient categories, on which immunoassays are known to be design-dependent flawed, e.g. pregnant females or patients with nonthyroidal illness (NTI), were purposely excluded as this would jeopardize the use of a uniform recalibration basis unless the design was adapted/optimized by the manufacturer [23,24]. As previously, the results are reported without identification of the assay/manufacturer [12,13,14,18], which was a carefully thought-out decision to protect the integrity and long-term objectives of the project (see the explanation in the online suppl. material). Note, however, that it is the intention to disclose the identity upon publishing the final standardization/harmonization study.

### Status of Standardization

The status of standardization/harmonization was judged by applying an arbitrary limit. Immunoassay performance within 10% from a hierarchically higher reference (the ED ID-LC/tandem MS cRMP for FT4, the APTM for TSH) was considered state of the art [13,18]. For FT4, it was exceeded by far by all immunoassays. In the mid- to high concentration range, they all were strongly negatively biased. In contrast, for concentrations <9 pmol/l, six assays tended to positive deviations. In addition, the data showed a considerable between-assay discrepancy. This was inferred from a difference between the assays that gave the lowest and highest FT4 results in the order of approximately 30% (assays K and M in the range of 9-27 pmol/l, F and B in the range >27 pmol/l) to even approximately 90% (assay B and E in the range <9 pmol/l). This reconfirmed that, currently, FT4 measurement results outside the range of one assay are well within that of another, which can confuse clinicians when they do not consider assay-specific reference intervals.

For TSH, concentration-dependent differences to the APTM and interassay were also observed; however, they remained for the greater part within 10%. In the mid-concentration range, the assays even compared fairly well since only two deviated by more than 10%. In the pathophysiological ranges (<0.5 and >5 mIU/l, respectively), the numbers were somewhat higher (5 and 4, respectively). Nevertheless, the discrepancy between the most extreme assays was considerable since one (assay I) negatively deviated by −21 to −33%, while another (assay K) was positively biased by 8-12%. Other assays switched in a concentration-dependent manner from a negative to a positive bias. These observations emphasize that harmonization is necessary, especially in light of current clinical practice discussions regarding decision limits in absolute values, e.g. 4.5 mIU/l in adults and or 2.5 mIU/l in pregnancy [25,26,27,28]. Consequently, until harmonization is reached, journals should emphasize the need to identify the assay used to generate data in clinical studies.

The excellent correlation of each of the TSH assays to the APTM (correlation coefficients at least 0.995) supports the argument that current immunoassays are ‘glycosylation blind’, in spite of the evidence that both core and terminal glycosylation alter epitope expression in TSH [19]. A second argument against pathophysiological-specific glycosylation as a potential cause of measurement discordance is that for most assays there was neither an obvious indication of sample-related effects (see online suppl. fig. S2), nor of a different performance on particular sample groups (apart from a calibration issue in the low concentration range for three assays).

In general, the study strengthened our past findings that, standardization/harmonization would be of great benefit for both FT4 and TSH, especially in the low concentration range.

### Success of Recalibration

For FT4, recalibration markedly improved the agreement of the assays with the cRMP (fig. 1c), although it appears that for most assays recalibration in the low concentration range can be improved (online fig. S1).

For TSH, on the other hand, recalibration mainly centered the distribution of the assay differences around zero (fig. 1d, online suppl. fig. S2). In general, the success of recalibration for the entire concentration range was good; however, for certain assays (F, G, and B in online suppl. fig. S2), it was poor, <0.30 mIU/l, with differences up to approximately −80%. For assay C, a peculiar shape in the difference plot was observed after recalibration, most probably due to the use of distinct recalibration functions for low and high concentrations. In fact, this phenomenon was already present in the initial data, and recalibration did not correct it.

Overall, for most recalibrated FT4 and TSH assays, the remaining total error is nearly entirely due to random error components. An additional measure of the benefits of recalibration is the interassay CV (fig. 1e, f). For both analytes, it decreases significantly below 10% for the majority of the samples (apart from those with low concentrations), indicating the improved closeness of results.

Although standardization/harmonization is technically feasible, at least for the clinical samples used in this study, there is still a long road to go before implementation will become effective. Besides some straightforward technical issues, such as the establishment of infrastructure to sustain standardization/harmonization, it will be essential for the C-STFT to openly collaborate with a broad spectrum of representatives with a stake in ensuring reliable laboratory testing for optimal/efficient management of patients with thyroid disease. These representatives should include laboratory directors, regulatory agencies, professional societies, pharmaceutical companies, and of course physicians and their patients. They will, for example, have to agree on the best point in time for global implementation (all assays/all manufacturers), and discuss whether, after implementation, a broader spectrum of samples should be used to study the influence of certain clinical factors on assay performance, e.g. glycoheterogeneity and NTI. This might help to decide which patient categories should, if any, be deemed for measurement with current immunoassays (e.g. as recommended by the National Academy of Clinical Biochemistry for NTI and others [29,30]), and/or call for new assay generations with dedicated designs. Medical journals and professional societies should serve as additional vehicles.

Indeed, to avoid interpretation errors, the impact of recalibration on the numerical values obtained by individual immunoassays (thus, on their reference interval or decision limits) will require transition with caution. This study showed that particularly for FT4 assays the impact will be huge. The current calibration set point will increase between approximately 15 and 50% (in the mid- to high concentration range). For example, for a specific sample before recalibration, assays K and M report approximately 10.5 pmol/l and 15.8 pmol/l, respectively; afterwards both would report approximately 19 pmol/l. In contrast, for TSH the impact will not be as dramatic since only 3-4 assays will be significantly affected (1 over the whole concentration range, 2 in the low range, and 1 in the high range). From this perspective, C-STFT considers the establishment of the physician/laboratory-interface of particular importance within the relationship of all stakeholders. The endocrine societies and the IFCC should establish a joint committee to address these issues.

## Disclosure Statement

All authors have no disclosures to make, apart from Frank A. Quinn, who reports employment by Abbott and stock ownership in Abbott.

## Figures and Tables

**Table 1:** Characteristics of the FT4 and TSH serum panel

	FT4 panel	TSH panel
Number of samples	74	94

Target setting	ED ID-LC/tandem MS	APTM

Concentration range	3–77 pmol/l	0.04–80 mIU/l

Inclusion criteria	Individuals were at least 18 years old and competent to give informed consent, as considered by the physician, study nurse or other health care professional interviewing the patient	
	Individuals being evaluated for a thyroid disorder and classified into one of the following groups (if possible evenly distributed):	
	
	D: hyperthyroid (n = 30)	A: hyperthyroid (n = 30)
	Patients with FT4 values >28 pmol/l up to 40 pmol/l^a^	A1: 10 patients with suppressed TSH, around 0.01 mIU/l
		A2: 10 patients with TSH values between 0.01 and 0.1 mIU/l
		A3: 10 patients with TSH values between 0.1 and 0.3 mIU/l^a^
	
	E: euthyroid (n = 120)	B: euthyroid (n = 30)
	Patients with FT4 values between 10 and 28 pmol/l^a^	Patients with TSH values between 0.3 and 3.0 mIU/l^a^
	
	F: hypothyroid (n = 30)	C: hypothyroid (n = 40)
	Patients with FT4 values between 3 and 10 pmol/l^a^	C1: 20 patients with TSH values between 3.0 and 50 mIU/l^a^
		C2: 20 patients with TSH values >50 mIU/l up to 100 mIU/l
	Donors treated for thyroid dysfunction were included, provided information on the type of treatment and start of the treatment was available	
	Note: samples were measured for their endogenous analyte concentration, hence subjects treated with L-thyroxine were only included in the TSH panel and vice versa for patients treated with recombinant TSH	

Exclusion criteria	Individuals previously enrolled into this clinical study	
	Individuals diagnosed with a severe NTI, defined as a state of dysregulation where levels of T3, T4, FT3, and/or FT4 are abnormal although the thyroid gland does not appear to be dysfunctional	
	Individuals with known pregnancy	
	Patients not meeting the established inclusion criteria	

a These values are indicative because they depend on the measurement range and the reference interval of the assay used to evaluate the FT4 or TSH status.

**Table 2:** Study participants and assays

Assay manufacturer	Assay	Analyte
Abbott Diagnostics (Abbott Park, Ill., USA)	Architect i2000SR	FT4 and TSH
Beckman Coulter, Inc. (Brea, Calif., USA)	Access 2	FT4 and TSH
bioMérieux s.a.	VIDAS FT4	FT4
(Marcy-l'Etoile, France)	VIDAS TSH & TSH3	TSH
DiaSorin S.p.A. (Saluggia, Italy)	Liaison	FT4 and TSH
Ortho-Clinical Diagnostics (Buckinghamshire, UK)	VITROS Immunodiagnostic Systems (ECiQ and 3600)	FT4 and TSH
Roche Diagnostics GmbH (Mannheim, Germany)	Elecsys	FT4 and TSH
Siemens Healthcare Diagnostics Inc.	ADVIA Centaur	FT4
(Deerfield, Ill., USA)	ADVIA Centaur TSH3-UL	TSH
	Dimension RxL	FT4 and TSH
	Dimension EXL with LOCI module	FT4
	Dimension EXL with LOCI module (3rd generation)	TSH
	Dimension Vista 1500	FT4 and TSH
	IMMULITE 2000	FT4
	IMMULITE 2000 (Third Generation TSH)	TSH
Tosoh Corporation (Tokyo, Japan)	AIA-2000 (ST AIA-PACK)	FT4 and TSH

**Table 3:** Assay bias (mean %) vs. the ED ID-LC/tandem MS cRMP or APTM (before and after recalibration)

FT4 assay	Before recalibration			After recalibration		
	<9 pmol/l	9–27 pmol/l	>27 pmol/l	<9 pmol/l	9–27 pmol/l	>27 pmol/l
M	38	−14	−33	17	−0.9	−3.5
E	62	−18	−43	66	−7.8	−6.1
G	4.6	−20	−34	−8.5	1.7	−1.6
B	−28	−20	−21	−12	−1.5	−2.2
H	23	−22	−42	23	0.2	1.0
D	26	−23	−41	31	−0.1	1.4
I	−18	−24	−30	13	4.6	−3.6
C	−10	−25	−37	16.1	4.8	−6.9
A	−27	−26	−27	−9.2	−7.8	−8.6
L	5.7	−28	−45	−15	0.5	−1.2
J	−9.3	−29	−37	−5.6	3.2	−0.2
F	−27	−37	−48	−8.9	2.7	0.2
K	−15	−42	−45	19	1.9	2.1

TSH assay	Before recalibration			After recalibration		
	0.03–0.5 mIU/l	0.5–5 mIU/l	≤5 mIU/l	0. 03–0.5 mIU/l	0.5–5 mIU/l	≤5 mIU/l
I	−33	−23	−21	−10	0.8	−1.1
A	−7.7	−8.4	−17	−5.9	5.0	5.2
J	−10	−6.2	−2.3	4.3	1.4	−0.4
M	−8.7	−5.4	−0.3	−8.3	−6.7	−3.3
L	−3.6	−3.8	−6.8	−2.0	−0.7	−0.5
F	−19	−1.9	−0.3	−19	−2.7	1.8
H	−7.4	−1.9	7.4	−2.8	0.0	−0.3
N	−3.7	−1.4	3.5	−0.8	−3.0	−0.3
D	4.4	0.7	−1.2	5.6	1.4	−0.2
G	−19	1.2	4.6	−21	−0.3	3.8
E	7.6	7.7	7.6	−2.0	−0.9	−1.9
K	12	8.3	12	−3.6	−1.5	−1.4
C	−2.1	9.4	8.4	−10	1.5	−1.2
B	4.4	11	−17	−15	1.4	6.0

Biases before recalibration sorted in ascending order (FT4 in the concentration range 9–27 pmol/l and TSH in the range 0.5–5 mIU/l).
